# Genome survey sequencing of the Caribbean spiny lobster *Panulirus argus*: Genome size, nuclear rRNA operon, repetitive elements, and microsatellite discovery

**DOI:** 10.7717/peerj.10554

**Published:** 2020-12-17

**Authors:** J. Antonio Baeza

**Affiliations:** 1 Department of Biological Sciences, Clemson University, Clemson, SC, USA; 2 Departamento de Biologia Marina, Universidad Catolica del Norte, Coquimbo, IV Region, Chile; 3 Smithsonian Marine Station at Fort Pierce, Smithsonian Institution, Fort Pierce, FL, USA

**Keywords:** Genome survey, Repetitive elements, Crustacea, Nuclear ribosomal operon

## Abstract

**Background:**

*Panulirus argus* is an ecologically relevant species in shallow water hard-bottom environments and coral reefs and target of the most lucrative fishery in the greater Caribbean region.

**Methods:**

This study reports, for the first time, the genome size and nuclear repetitive elements, including the 45S ribosomal DNA operon, 5S unit, and microsatellites, of *P. argus*.

**Results:**

Using a *k*-mer approach, the average haploid genome size estimated for *P. argus* was 2.17 Gbp. Repetitive elements comprised 69.02% of the nuclear genome. In turn, 30.98% of the genome represented low- or single-copy sequences. A considerable proportion of repetitive sequences could not be assigned to known repeat element families. Taking into account only annotated repetitive elements, the most frequent belonged to Class I-LINE which were noticeably more abundant than Class I-LTR-Ty- 3/Gypsy, Class I-LTR-Penelope, and Class I-LTR-Ty-3/Bel-Pao elements. Satellite DNA was also abundant. The ribosomal operon in *P. argus* comprises, in the following order, a 5′ ETS (length = 707 bp), ssrDNA (1,875 bp), ITS1 (736 bp), 5.8S rDNA (162 bp), ITS2 (1,314 bp), lsrDNA (5,387 bp), and 3′ ETS (287 bp). A total of 1,281 SSRs were identified.

## Introduction

The order Decapoda is one of the most species-rich crustacean clades (De Grave et al., 2009). In this clade, clawless lobsters (infraorder Achelata) differ markedly in their morphology, behavior, and ecology (De Grave et al., 2009; Lozano-Fernandez et al., 2019). Clawless lobsters lack chelae on the first pair of pereopods and also share a unique larval type; the long-lived phyllosomata. Many species attain large body sizes and relatively high population densities, and thus, are the target of major fisheries in all subtropical and tropical oceans (Holthuis, 1991).

The Caribbean spiny lobster *Panulirus argus* (Latreille, 1804) is an ecologically relevant species in coral reefs (Higgs, Newton & Attrill, 2016) and target of the most profitable fishery in the greater Caribbean region (Holthuis, 1991). *Panulirus argus* inhabits the western Atlantic Ocean from North Carolina in the northern hemisphere to southern Brazil in the southern hemisphere (Holthuis, 1991). The life history of *P. argus* is well understood (Baeza et al., 2016; Baeza, 2018, and references therein). Adult females can produce 2–4 clutches of eggs per year (Maxwell et al., 2009). Fecundity increases with female body size and ranges between 100,000 and 750,000 eggs per female (Baeza et al., 2016). After completion of embryo development and hatching of larvae, 10 consecutive planktonic stages succeed one another (Goldstein et al., 2008). These planktotrophic phyllosomata larvae can spend 4–18 months suspended in the water column (Goldstein et al., 2008). The 10th larval stage undergoes a metamorphosis offshore, turning into a fast-swimming, lecithotrophic, short-lived (2–4 weeks) “puerulus” post-larval stage with morphology similar to that of juvenile and adult benthic lobsters, but almost devoid of coloration (Phillips et al., 2006). Pueruli actively swim from the open ocean to shallow coastal habitats, where they settle in vegetated habitats (Baeza, Childress & Ambrosio, 2018, and references therein). A few days after settlement, pueruli molt and metamorphose into early benthic juveniles that resume feeding and dwell in the vegetation for several months (Lewis, Moore & Babis, 1952; Behringer et al., 2009). Later, juveniles emerge, become social, and are often found sharing crevice shelters with other juveniles as well as subadult lobsters (Childress & Herrnkind, 1997). As lobsters near maturity (~1.5 years post-settlement), they migrate to join the adult population on coral reefs, where they mate and reproduce (Childress & Herrnkind, 1996; Bertelsen & Hornbeck, 2009; Baeza et al., 2016). *P. argus* is fully exploited or overexploited across its entire geographic range (Holthuis, 1991).

A few genomic resources exist for *P. argus* (Kozma et al., 2018; Baeza, 2018; Baeza, Umaña-Castro & Mejia-Ortiz, 2019; Baeza & MacManes, 2020; Subramaniam et al., 2020). The mitochondrial genome of *P. argus* has recently been assembled and analyzed in detail (Baeza, 2018). Also, the heart + hemolymph (Baeza & MacManes, 2020) and nervous system (Kozma et al., 2018) transcriptomes have been assembled and annotated. Lastly, a SNP panel developed using 2bRAD-sequencing has been tested in order to explore meta-population connectivity throughout the greater Caribbean basin in this species (Baeza, Umaña-Castro & Mejia-Ortiz, 2019).

This study forms part of an effort to develop genomic resources in *P. argus* and congeneric species in the western Atlantic and Gulf of Mexico. Using a low-pass short read next generation sequencing approach, I have estimated the genome size using an *in-silico* approach, explored repetitive elements in the nuclear genome, assembled ribosomal RNA genes, including the 45S operon and 5S unit, and discovered microsatellites.

## Materials and Methods

### Sampling of *Panulirus argus* and DNA extraction

Field collection was approved by FWCC (permit number: SAL-11-1319-SR). The studied specimen was collected as previously described in Baeza (2018). In brief, one adult female of *P. argus* was collected in July 2017 by hand while SCUBA diving from a patch reef on the ocean side of Long Key (N24°49′26″; W80°48′48″), Florida, USA and transported alive to Clemson University, Clemson, SC. In the laboratory, the specimen was maintained in a 500 L circular polyethylene container. Muscle was extracted from a pereiopod, and the tissue was immediately snap-frozen within a 50 ml centrifuge tube located inside a 3 L plastic ice chest containing dry ice blocks (−78.5 °C). Within an hour of tissue extraction, the sample was transported to OMEGA Bioservices (Norcross, GA, USA).

### Library preparation, and sequencing

Total genomic DNA was extracted as described in Baeza (2018) from the muscle tissue using the OMEGA BIO-TEK^®^ E.Z.N.A.^®^ Blood and Tissue DNA Kit following the manufacturer’s protocol. DNA concentration was measured using the QuantiFluor dsDNA system on a Quantus Fluorometer (Promega, Madison, WI, USA). A Kapa Biosystems HyperPrep kit (Kapa Biosystems, Wilmington, MA, USA) was used for whole genome library construction. Briefly, 1 µg of genomic DNA was fragmented using a Bioruptor sonicator (Diagenode, Denville, NJ, USA). DNA fragment ends were repaired, 3′ adenylated, and ligated to Illumina adapters. The resulting adapter-ligated libraries were PCR-amplified, Illumina indexes added and pooled for multiplexed sequencing on an Illumina HiSeq X10 sequencer (Illumina, San Diego, CA, USA) using a pair-end 150 bp run format.

A total of 439,834,692 PE reads used in this study are available in the short read archive (SRA) repository (accession number SRR13036344) at GenBank.

### Genome size of *Panulirus argus*

Contaminants, low quality sequences (Phred scores < 20), and Illumina adapters were removed using the software fastp using default parameters (Chen et al., 2018), leaving 415,398,624 high quality reads for genome size estimation with the software Jellyfish-2 using *k*-mer = 21 (Marçais & Kingsford, 2011) and the server GenomeScope 2.0 (Ranallo-Benavidez, Jaron & Schatz, 2020).

### Repetitive elements in the genome of *Panulirus argus*

Discovery, characterization, and quantification of repetitive elements in the genome of *P. argus* were conducted as described in Baeza (2020) using the workflow RepeatExplorer 2.3.8 (Novak et al., 2013) in the platform Galaxy (http://repeatexplorer.org/). After a first clustering step, reads are assembled into contigs using the program CAP3 (Huang & Madan, 1999) and annotated using the internal Metazoa version 3.0 repeat dataset included in the package. All other parameters in RepeatExplorer were set to default values. The pipeline RepeatExplorer was originally developed for analyzing plants, and thus, annotation is known to be more efficient for plants than animals (Novak, Neumann & Macas, 2020). Still, RepeatExplorer is efficient in analyzing repeat composition and abundance of animal genomes with low coverage Illumina PE sequences (Novak et al., 2013, 2020).

### Nuclear ribosomal operon/tandem repeat in *Panulirus argus*

The nuclear ribosomal operon, that codes for the large and small nuclear rRNA genes (18S or ssrDNA, 28S or lsrDNA), the 5.8S rDNA gene, two internal transcribed spacers (ITS1 and ITS2), and two external transcribed spacers (5′ ETS and 3′ ETS), in the genome of *P. argus* was characterized using the contigs assembled by the program CAP3 that were annotated as nuclear repetitive ribosomal DNA by RepeatExplorer. Each contig (*n* = 38) was blasted against the non-redundant (nr) nucleotide NCBI database as well as Dfam (Hubley et al., 2016) and Rfam (Kalvari et al., 2018). Contigs not matching crustacean ribosomal sequences with *E*-values < 1e−6 were discarded (*n* = 3), the remaining contigs were aligned with Muscle (Edgar, 2004) with default parameters as implemented in MEGA (Kumar et al., 2018) and the assembly was curated manually. The exact coding positions of the 18S and 28S nuclear rDNAs and the boundaries of the 5′ and 3′ ETSs were determined using RNAmmer with default parameters (Lagesen et al., 2007). The exact coding positions of the 5.8S nuclear rDNA and the boundaries of the ITS1 and ITS2 were determined using ITSx (Bengtsson-Palme et al., 2013). The Neural Network Promoter Prediction (NNPP) tool (Reese, 2001) available at the Berkeley Drosophila Genome project (http://www.fruitfly.org/seq_tools/promoter.html) was used with default parameters to determine the putative position of a promoter in the 5′ ETS. Lastly, the PE reads were mapped to the newly assembled rDNA operon using bowtie2 2.4.2 (Langmead & Salzberg, 2012) with default parameters to determine coverage.

### Microsatellite discovery in *Panulirus argus*

Simple Sequence Repeats in the genome of *P. argus* were discovered as described in Baeza (2020) with the pipeline Pal_finder v0.02.04.08 in the platform Galaxy (https://palfinder.ls.manchester.ac.uk) (Griffiths et al., 2016). After scanning for the existence of SSRs (Simple Sequence Repeats) (di-, tri-, tetra-, penta-, and hexa-nucleotide motif repeats) in previously filtered Illumina PE short reads, PCR primers were developed with the program Primer3 (Untergasser et al., 2012). Also, the software PANDAseq (Masella et al., 2012) was used to assemble paired-end reads and confirm that the primer sequences were present in a high-quality assembly based on the available reads. A minimum of 5 repeats were requested for the program pal_finder to select 2-mer SSRs and a minimum of 6 repeats to select SSRs with 3, 4, 5, and 6 repeat motifs.

## Results and Discussion

### Genome size of *Panulirus argus*

**Figure 1:**
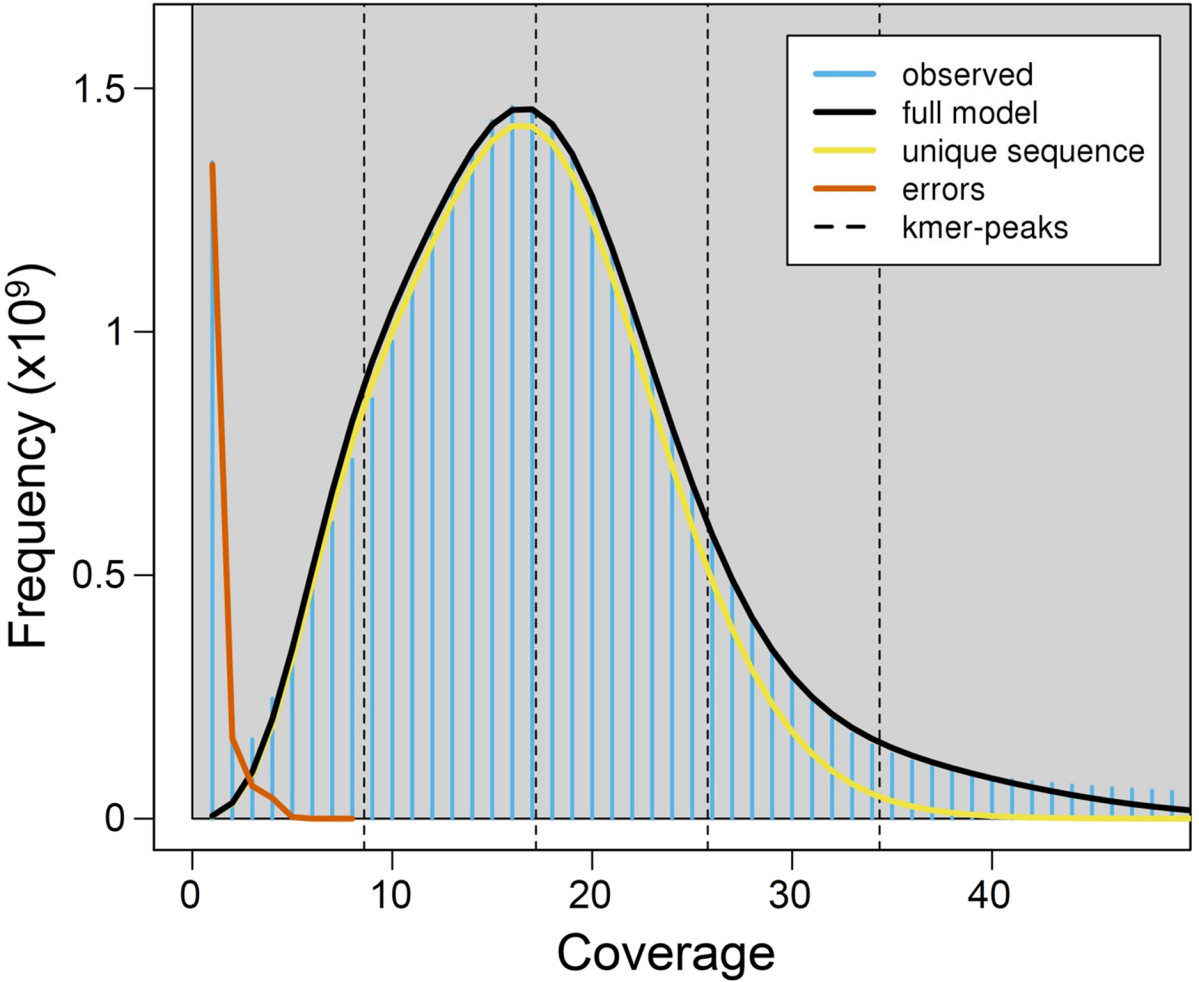
*K*-mer (*k* = 21) spectrum analysis to estimate genome size in the Caribbean spiny lobster *Panulirus argus*. The plot shows major peaks and a high frequency of putative error *k*-mers with coverage <10×. The abscissa (*x*-axis) is depth of coverage and the ordinate (*y*-axis) is the proportion that represents the frequency at that depth divided by the total frequency of all depths.

Using a *k*-mer approach, the average haploid genome size estimated for *P.argus* was 2.17 Gbp, with a relatively low level of genome heterozygosis (het. = 1.48%), and a moderately high unique genome content (64.4%) (Fig. 1). Estimates of heterozygosis varied between 0.0039 and 0.0048 with an average (±SD) of 0.0042 (±0.0002) in ten different individuals of *P. argus* as reported by a previous study that used 2b-RAD sequencing (Baeza, Umaña-Castro & Mejia-Ortiz, 2019).

Genome size previously estimated for *P. argus* heart cells using static cell fluorometry is 5.55 Gbp (Jimenez et al., 2010). Genome size is moderately variable in the family Palinuridae, ranging from 3.15 Gbp in the Pink spiny lobster *Palinurus mauritanicus* to 5.55 Gbp in *P. argus* (animal genome size database (http://www.genomesize.com/), consulted 11/20/2020). The observed difference in genome size estimated for *P. argus* when calculated using a k-mer approach and cell fluorometry (Jimenez et al., 2010) might be due to the relatively large portion of repetitive elements in the nuclear genome of this species (see below). Repetitive elements are known to bias genome size estimations downwards when they account for a large portion of a genome (Baeza, 2020 and references therein). Furthermore, the large genome size estimated for *P. argus* (>2Gbp) implies that a combination of short and long-reads (i.e., PacBio, ONT) will likely be required for assembling a high quality (i.e., chromosome level) genome in this species. Given their length (~10–20 kbp—Jain et al., 2018), long reads can resolve repetitive genomic regions that otherwise are reconstructed with much lower resolution by short reads (Jain et al., 2018). In turn, short reads improve assembly accuracy given that their error rate is lower than that of long reads (Rhoads & Au, 2015; Rang, Kloosterman & De Ridder, 2018).

### Repetitive elements in the genome of *Panulirus argus*

**Figure 2:**
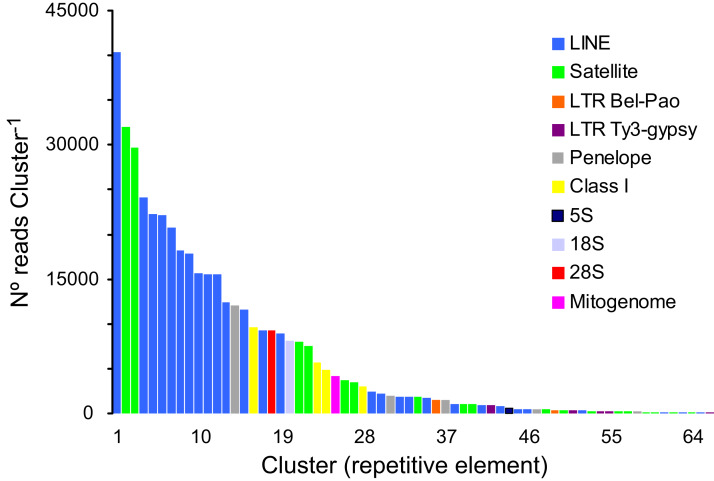
Size distribution and repeat composition of annotated clusters generated by similarity based partitioning of in the Caribbean spiny lobster *Panulirus argus*. Bars are colored according to the type of repeat present in the cluster, as determined by the similarity search in RepeatExplorer2.

A total of 166,576 clusters contained 69.02% of all analyzed reads (a sub-sample of 1,220,482 reads). In turn, 30.98% of the analyzed reads represented, putatively, low- or single-copy sequences in the genome of *P. argus*. The proportion of reads representing the most abundant repetitive elements in the genome of *P. argus* was 37.96% (in the top 127 clusters). Importantly, a large portion of the repetitive elements families (*n* = 63 clusters, 63,923 reads) were reported as “unclassified” by RepeatExplorer given that they could not be assigned to known repeat families. The above suggests that abundant new repetitive elements will be discovered by future studies focusing on the “repeatome” of *P. argus* as well as other crustaceans. Taking into account only annotated clusters, the most common repetitive elements belonged to Class I-Long Interspersed Nuclear Element (LINE) (*n* = 28 clusters, 269,194 reads), which were considerably more abundant than Class I-LTR-Penelope elements (*n* = 5, 16,095 reads), Class I-LTR-Ty-3/Bel-Pao elements (*n* = 2, 1,887 reads), and Class I-LTR-Ty-3/Gypsy elements (*n* = 5, 1,793 reads) (Fig. 2). Seventeen clusters were classified as Satellite DNA (82,666 reads). One cluster each was classified as 45S/28S (9,230 reads), 45S/18S (8,116 reads), and 5S (558 reads, and one assembled sequence of 4,161 bp in length (studied in detail below)) ribosomal DNA, respectively. RepeatExplorer also assembled the complete mitochondrial genome of *P. argus* (15,739 bp) that represented one cluster containing 4,230 reads. A detailed analysis of purifying selection and phylogenomic informativeness of protein coding genes in the mitochondrial genome of *P. argus* (using a smaller dataset compared to that used in this manuscript) has recently been published (Baeza, 2018).

Overall, this analysis revealed that a large part of the repeat element clusters represent various families of LINEs and Satellite DNA in the genome of *P. argus*. Repetitive elements such as LINEs and LTRs have been shown to account for a large portion of the assembled genome in the few decapod crustaceans in which the repeatome has been explored (Tang et al., 2020 for the Chinese mitten crab *Eriocheir japonica sinensis*).

### Nuclear ribosomal operon/tandem repeat in *Panulirus argus*

**Figure 3:**
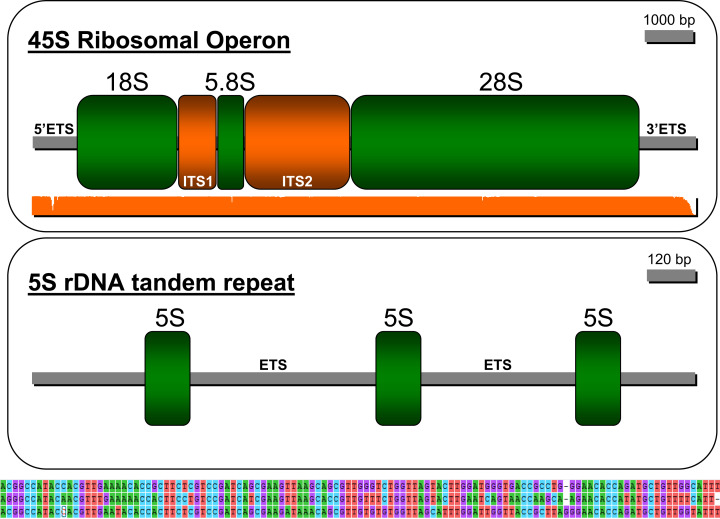
Depiction of the 45S ribosomal operon (above) and 5S unit tandem repeat (below) in the Caribbean spiny lobster *Panulirus argus*. The three sequences immediately below the depiction of the 5S tandem repeat are the three similar but different 5S genes.

The strategy employed in this study permitted reconstructing the ribosomal operon in *P. argus* (GenBank accession: MW252173) that comprises, in the following order, a 5′ ETS (length = 707 bp), ssrDNA (1,875 bp), ITS1 (736 bp), 5.8S rDNA (162 bp), ITS2 (1,314 bp), lsrDNA (5,387 bp), and 3′ ETS (287 bp (likely partial sequence)) (Fig. 3). The NNPP tool suggested the putative existence of a 50 bp promoter 5′-GTT GTT CTC TAA ATG ATA ACG CAC TAT CCC GGG GCG GCG C *A*G GAG CCG TT-3′ (score 0.8), upstream of the 18S rDNA gene starting at position 162 of the assembled sequence. Note that the italic and bold nucleotide in the sequence above is the predicted transcription initiation site (TIS). The presence of a putative terminator (Sal box alike sequence: 5′-AGG TCG ACC AG[T/A] [A/T]NT CCG-3′, see Kuhn et al. (1988)) was explored downstream of the 28S gene but not found likely because the 3′ EST was only partially assembled. Interestingly, the motif 5′-AGG TCC GGA CAT ATG GTT CCG-3′ (similar to the Sa1 box) was found starting at position 9391 of the assembled sequence, ~350 bp upstream of the 28S gene end as determined by RNAmmer. S1 nuclease mapping, among additional bench-work, is required to confirm this *in-silico* predictions. Mapping of the trimmed PE reads on the newly assembled rRNA operon indicated an overall alignment rate of 1.28%. Seven nuclear rDNA sequences available in Genbank were a close match to the de novo assembled rDNA operon of *P. argus* (*p*-distance 18S rDNA partial sequence (1,872 bp in length, accession number: U019182) = 0.012; *p*-distance 18S rDNA complete sequence (1,872 bp, AY743955) = 0.014; *p*-distance 18S rDNA partial sequence (1,872 bp, AY743955) = 0.009; *p*-distance 5.8S rDNA partial sequence (385 bp, AY210832) = 0.021; *p*-distance 28S rDNA partial sequence (941, AY210835) = 0.02; *p*-distance 28S rDNA partial sequence (1,582 bp, AY210834) = 0.008; *p*-distance 28S rDNA partial sequence (2,282 bp, AY210833) ≤ 0.001).

The sequence assembled by CAP3 and annotated as the 5S rDNA gene (GenBank accession: MW251820) in RepeatExplorer comprised three tandem-organized copies of the 5S unit as indicated by the software RNAmmer. The three copies in the tandem repeat assembly were not identical but all were a close match to each other (from 5′ to 3′ in the assembly, 1st vs 2nd copy *p*-value = 0.214; 2nd vs 3rd copy *p*-value = 0.111; 1st vs 3rd copy *p*-value = 0.248, see Fig. 3 lower panel). The non-transcribed spacer (NTS) sequence between the first and second and the second and the third 5S units were 1,319 and 1,892 bp in length, respectively. Various SSRs but no transposable elements were found in these NTS sequences.

Studies on the structure and organization of both the nuclear ribosomal operon and the 5S rDNA gene are most rare in decapod crustaceans, including lobsters (Vierna et al., 2013 and references therein, but see for example, the crab *Eriocheir sinensis* (Yu et al., 2010) for one of a few exceptions). In the higher eukaryotes, including arthropods, these two distinct gene families, the 45S and 5S ribosomal DNA genes, are organized in tandem arrays of a single, a few, or hundreds to thousands of copies that can be found on either one, a few, or several chromosome pairs (Vierna et al., 2013). Two distinct classes of 5S rDNA tandem repeats has been observed in some species and, depending upon the studied group, the same gene can or cannot be linked to other repetitive noncoding RNA or protein-coding gene families (Vierna et al., 2013). The newly described genomic organization of the ribosomal operon and 5S rDNA unit will serve as the base for future studies examining the evolution (i.e., concerted evolution vs birth-and-death models—Nei & Rooney, 2005) of these most relevant genomic elements in lobsters and other decapod crustaceans. This new information will also facilitate the development of new low pass sequencing + gene-targeted phylogenomic approaches (as an alternative or addition to for example, anchored hybrid enrichment (Lemmon, Emme & Lemmon, 2012) and ultra-conserved elements (Faircloth et al., 2015)) to study the genealogical relationships within lobsters as well as among lobsters and other decapod crustaceans.

### Microsatellite discovery in *Panulirus argus*

A total of 1,281 suitable SSR primer pairs (*N* = 1012, 212, 48, 9, and 0 for 2mer, 3mer, 4mer, 5mer and 6mer SSRs motifs, respectively) were identified using the most stringent filtering options for finding SSRs in pal_finder (Supplemental Materials, File S1). A single previous study using a limited number of SSRs (*n* = 11) suggested a pattern of chaotic patchiness for *P. argus* in the Caribbean Sea (Truelove et al., 2017). Studies combining a subset of the SSRs identified here (after further development), nuclear SNPs (Baeza, Umaña-Castro & Mejia-Ortiz, 2019), and mitochondrial genomes (Baeza, 2018) can be used to assess population genetic/genomic connectivity in *P. argus* across its range of distribution. Furthermore, these new resources will aid in answering questions relevant for the conservation and fisheries management of this species, including but not limited to determining kinship aggregations within populations, effective population sizes (Baeza, Childress & Ambrosio, 2018), recent migration rates, source and sink meta-population dynamics, as well as local adaptation (Oleksiak & Rajora, 2020).

## Conclusion

This study developed genomic resources for the Caribbean spiny lobster *P. argus*, an ecologically relevant species in coral reefs and a target of the most profitable fishery in the greater Caribbean. Using low-pass short read Illumina sequencing, the genome size was estimated, nuclear repetitive elements were identified, partially classified, and quantified, and the ribosomal RNA operon and 5S rRNA DNA unit were assembled. A large set of SSRs was also detected. This information will contribute to the better understanding of meta-population connectivity and putative genomic mechanisms involved in the acclimatization and adaptation to climate change in *P. argus*.

## Supplemental Information

10.7717/peerj.10554/supp-1 Supplemental Information 1 Microsatellite primers discovered after a stringent search with the software palfinder in the Caribbean spiny lobster *Panulirus argus*.

10.7717/peerj.10554/supp-2 Supplemental Information 2 45S ribosomal rna dna operon and 5S ribosomal rna dna tandem repeat in the Caribbean spiny lobster *Panulirus argus*.

## Additional Information and Declarations

### Field Study Permissions

The following information was supplied relating to field study approvals (i.e., approving body and any reference numbers): Field collection was approved by FWCC (permit number: SAL-11-1319-SR).

### Data Availability

The following information was supplied regarding data availability: Sequences described here are accessible via GenBank: MW252173 and MW251820, BioProject ID: PRJNA453553. Sequences described are also available in the Supplemental Files.
